# Gross Total Resection and Recurrence-Free Survival in Diffuse Gliomas: A Single-Center Cohort Study from Eastern Europe

**DOI:** 10.3390/clinpract16080155

**Published:** 2026-08-20

**Authors:** Teodor Cristian Blidaru, Marius Cristian Zaharia, Dan Mitrea, Raluca Maria Marin, Natalia Blidaru, Manuela Ghica, Maria Sinziana Matei, Raluca Papacocea, Stefan Strilciuc, Dragos Nicolae Garofil

**Affiliations:** 1 Faculty of Medicine, Carol Davila University of Medicine and Pharmacy, 020021 Bucharest, Romania; 2 Neuroaxis Neurology Clinic, 040215 Bucharest, Romania; dan.mitrea@neuroaxis.ro; 3 Faculty of Pharmacy, Carol Davila University of Medicine and Pharmacy, 020021 Bucharest, Romania; 4 Department of Genomics, Medfuture Institute for Biomedical Research, Iuliu Hatieganu University of Medicine and Pharmacy, 400012 Cluj-Napoca, Romania

**Keywords:** diffuse glioma, extent of resection, gross total resection, multivariable analysis, neuro-oncology, postoperative MRI, recurrence-free survival, residual tumor

## Abstract

**Background/Objectives:** The independent contribution of extent of resection (EOR) to early recurrence in diffuse gliomas remains debated, as crude analyses are confounded by tumor biology, and real-world data from Eastern European centers are scarce. **Methods:** We retrospectively studied 283 consecutive adults with surgically treated diffuse gliomas (2021–2024) at a Romanian tertiary center. Primary endpoints were recurrence-free survival (RFS) at 1 and 2 years, evaluated as landmark endpoints; secondary endpoints were overall recurrence, reoperation, and reirradiation. Clinical, histopathological, surgical, imaging, and treatment variables were analyzed with complete-case Firth-penalized logistic regression. **Results:** Among evaluable patients, 70.6% (132/187) were recurrence-free at 1 year and 64.2% (120/187) at 2 years; overall recurrence occurred in 53.7% (139/259). After adjustment, gross total resection (GTR) was independently associated with higher odds of remaining recurrence-free at 1 year (OR 12.37, 95% CI 3.23–52.58; *p* < 0.001) and 2 years (OR 10.56; *p* < 0.001), as was subtotal resection (STR) at both time points (OR 4.15, 95% CI 1.17–16.16, *p* = 0.027; and OR 3.93, *p* = 0.027), versus partial resection/biopsy. **Conclusions:** EOR was independently associated with early RFS, supporting maximal safe resection as a primary surgical objective. The landmark endpoints were not evaluable in 96 of 283 patients, almost all of whom underwent biopsy or partial resection, so the odds ratios indicate the direction of the effect and not its precise magnitude. Overall recurrence, evaluable in 91.5% of the cohort, showed a concordant gradient. External validation is needed.

## 1. Introduction

Diffuse gliomas are one of the most complex primary brain tumors due to their growth patterns of infiltration, intrinsic resistance to therapy, and, ultimately, recurrence, even in patients who received multi-modality therapy [1,2]. The WHO 2021 classification redefined them as biologically distinct entities on the basis of combined histological and molecular features, notably isocitrate dehydrogenase (IDH) mutation status and codeletion of chromosome arms 1p and 19q [1], improving prognostic stratification and treatment decision-making. However, the role of surgical excision remains unchanged as a first-line treatment modality.

Maximal safe surgical resection remains the primary treatment modality, being the only intervention that reliably produces immediate cytoreduction [2,3]; both the European Association of Neuro-Oncology (EANO) and international guidelines recommend it regardless of molecular subtype whenever it is feasible [2]. A consistent body of evidence, including large meta-analyses, supports an extent-dependent association between extent of resection (EOR) and both overall survival (OS) and recurrence-free survival (RFS), particularly in glioblastoma and IDH-mutant diffuse gliomas [3,4,5]. Volumetric imaging has extended this observation, showing that even small volumes of residual tumor on early postoperative magnetic resonance imaging (MRI) correlate with worse outcomes, which has motivated finer resection thresholds for distinguishing near-total from total macroscopic removal [3,6].

Outcome after surgery is nevertheless shaped by determinants other than EOR itself, notably tumor biology and the anatomical constraints that govern both resectability and prognosis. Retrospective comparisons of surgical and oncological outcomes therefore remain vulnerable to confounding and selection bias [7]. Inconsistent classification of EOR, ranging from surgeon-reported estimates to volumetric measurement, further limits comparability across studies [3,8]. The RANO resect group has recognized these issues, proposing standardized categories for EOR and emphasizing the incorporation of imaging-based measures of residual tumor when modeling outcome data [3,5].

OS has traditionally been the gold-standard endpoint in neuro-oncology [2], but RFS has gained attention as a clinically informative complement, since it captures early disease control and helps identify modifiable determinants of recurrence in heterogeneous cohorts [6,9,10]. A related question concerns postoperative imaging: early postoperative MRI studies have identified residual tumor presence and volume as prognostic factors that are not fully captured by categorical EOR classification [6,8], although the literature is conflicting on whether both should be entered jointly into multivariable models [5,6].

In this context, real-world data from single-center cohorts are crucial for validating the results from selective clinical trials [3,7]. Additionally, the literature is lacking with respect to data derived from neuro-oncology centers within Eastern Europe, particularly due to possible variances in patient characteristics, treatment pathways and access to molecular diagnostics [11,12,13].

Against this background, the present study has two primary aims, with an exploratory addition. First, we evaluate whether gross total resection (GTR) independently predicts recurrence-free survival at 1 and 2 years in a consecutive cohort of adults with diffuse gliomas treated at a Romanian tertiary neuro-oncology center between 2021 and 2024. Second, we examine the contribution of postoperative imaging-defined residual disease and other clinical and treatment-related variables to recurrence dynamics and to the secondary endpoints of overall recurrence, reoperation, and reirradiation. As an exploratory addition, we develop a recurrence-prediction nomogram with internal calibration and decision curve analysis, reported in Supplementary Materials. To our knowledge, this is one of the few multivariable evaluations of EOR-related recurrence dynamics integrating surgical, postoperative imaging, and clinical variables reported from an Eastern European tertiary neuro-oncology center. We claim no methodological novelty. The analysis is deliberately conventional, and the value of the study lies in the data it reports, which come from a region under-represented in the current glioma surgical literature.

## 2. Materials and Methods

### 2.1. Study Design and Ethical Approval

This study was designed as a single-center retrospective cohort analysis conducted at a tertiary neuro-oncology institution in Romania. The primary objective was to evaluate the impact of the EOR on RFS at 1 and 2 years following surgical treatment for diffuse gliomas, as well as the association with secondary clinical outcomes, including recurrence, reoperation, and reirradiation.

The study was conducted in accordance with the Declaration of Helsinki [14] and approved by the Research Ethics Subcommittee of Carol Davila University of Medicine and Pharmacy, Bucharest, Romania (approval number 35495/4 December 2025); details on ethics, consent, and conduct are provided in the back matter.

The study design, conduct, and reporting adhered to the Strengthening the Reporting of Observational Studies in Epidemiology (STROBE) guidelines for observational cohort studies [15]. The completed STROBE checklist is provided as Supplementary Table S1.

### 2.2. Patient Selection

Patients were identified from the surgical and pathology databases maintained by the institution. All consecutive adults who underwent surgery for a confirmed diagnosis of diffuse glioma between 2021 and 2024 inclusive were screened, and 283 patients were included in the study.

Patients were included in the study if they met the following criteria: (i) were of adult age (18 years or older); (ii) had a histological diagnosis of diffuse glioma; (iii) had undergone surgical treatment for that diagnosis at the study center, with a retrievable operative record; and (iv) had an early postoperative MRI available for assessment of residual tumor. Completeness of the surgical documentation and of subsequent follow-up was not used as an eligibility filter, since applying one would have selected the cohort on the very variables under study. Endpoint-specific evaluability, including the patients in whom the resection category or a given outcome could not be determined, is reported in Section 2.6 and in Table 1.

Patients who underwent non-curative or biopsy-only procedures were retained within the sample and classified with a reported resection rate of <90% so that the cohort would preserve its real-world composition. In 12 of the 283 patients the available surgical documentation did not support assignment to any one of the three resection categories defined in Section 2.3.3; these patients were retained so that the denominator reflects consecutive surgical practice, but they could contribute to no analysis in which extent of resection served as a stratifying or adjustment variable, and all percentages for extent of resection are accordingly calculated among the 271 patients with a documented category.

Diffuse gliomas were classified according to the WHO Classification of Tumors of the Nervous System (2021), which incorporated both histological and molecular characteristics whenever possible [1].

### 2.3. Data Collection and Variable Definition

Baseline characteristics were recorded at the time of diagnosis and comprised continuous variables (age and the pre- and postoperative Karnofsky Performance Index, KPI) and categorical variables (sex and initial clinical presentation); the individual variables and their definitions are set out in Section 2.3.1, Section 2.3.2, Section 2.3.3, Section 2.3.4 and Section 2.3.5. The KPI was included as a predictor variable because of its known prognostic ability in neuro-oncology [2].

#### 2.3.1. Clinical Variables

The following baseline clinical parameters were recorded: (i) age at diagnosis (continuous variable); (ii) sex (male/female); (iii) preoperative KPI, reflecting baseline functional status; (iv) postoperative KPI, recorded at discharge or early postoperative follow-up; (v) initial clinical presentation, categorized according to predominant symptomatology (e.g., seizures, focal neurological deficit, intracranial hypertension).

The KPI was included as a functional variable given its established prognostic relevance in neuro-oncology [2]. The KPI is a standardized clinician-assessed measure of functional impairment, scored from 0 to 100 in 10-point increments, with higher values reflecting preserved functional capacity and lower values indicating progressive dependency and disability. In this study, preoperative KPI was used as an indicator of baseline functional reserve before surgery, while postoperative KPI reflected the patient’s functional status after surgical treatment [16].

#### 2.3.2. Tumor Characteristics

Tumor characteristics included histological subtype, recorded in three categories (astrocytoma, oligodendroglioma, glioblastoma), and CNS WHO grade (2, 3, or 4), classified according to the WHO Classification of Tumors of the Central Nervous System (2021) [1]. In multivariable modeling, histological subtype and WHO grade were entered as separate covariates to provide independent estimates of their respective contributions to outcome. In this cohort the astrocytoma category comprises IDH-mutant astrocytoma (CNS WHO grades 2 to 4) together with tumors reported as diffuse astrocytoma NOS in the patients for whom IDH testing was not available; this composite category is abbreviated ASTRO in the figures, while OLIGO and GBM denote oligodendroglioma (IDH-mutant, 1p/19q-codeleted) and glioblastoma (IDH-wildtype), respectively.

Molecular characterization was partial and mirrored real-world testing availability in an Eastern European setting. Testing was carried out in the pathology laboratories serving the referring centers, each according to its own standard operating procedures. Because specimens were processed across several laboratories over the study period, a single uniform assay platform for IDH, ATRX and 1p/19q cannot be specified retrospectively. This heterogeneity of testing practice is itself a characteristic of the resource-constrained setting described here, and it is the reason the molecular data are presented descriptively only. IDH status was determined in 244 of 283 patients (86.2%; IDH-mutant in 123 and IDH-wild-type in 121 of tested cases), ATRX in 190 (67.1%) and Ki-67 in 233 (82.3%), whereas 1p/19q codeletion was assessed in only 66 (23.3%), and MGMT promoter methylation was not routinely tested during the study period; additional markers (TERT, CDKN2A/B, EGFR, H3 K27M) were available in fewer than 10% of cases. Because a complete, WHO 2021-concordant integrated molecular profile was achievable in only a minority of patients, molecular variables were not incorporated as covariates in the multivariable models; their distribution is reported descriptively and is not used for biological stratification. Tumor laterality and gross anatomical location were additionally recorded from the preoperative MRI report and were coded categorically; both were used as covariates in the exploratory secondary analyses of reoperation and reirradiation only (Supplementary Section S3).

#### 2.3.3. Surgical Variables

Extent of resection (EOR) was derived primarily from the operative report and the surgeon-reported resection percentage, and was corroborated, where available, by early postoperative MRI. EOR was classified into three categories: gross total resection (GTR; GTR100, removal of the entire visible tumor), subtotal resection (STR; GTR90–95) and partial resection or biopsy (PR; <90%). This three-tier scheme is broadly aligned with the categorical frameworks applied in recent population-based glioblastoma cohorts and with the RANO resect classification [17]. Because the primary EOR assignment was surgeon-reported and quantitative volumetric confirmation was not uniformly available, the presence of residual tumor on early postoperative MRI was additionally entered as a separate covariate to mitigate potential misclassification. EOR itself was not derived from a volumetric comparison of pre- and postoperative tumor volumes, as the RANO resect group recommends for prospective studies [3,5]: preoperative volumes were not measured as part of routine care during the study period, so a volumetric resection fraction could not be reconstructed retrospectively. Surgeon-reported EOR corroborated by early postoperative MRI therefore represents the finest resolution of exposure measurement attainable in this dataset. Biopsy and partial resection are not equivalent procedures and are not regarded as such here; they were combined into a single reference stratum because both are non-curative with respect to macroscopic disease, leaving measurable tumor in situ at the end of the operation, and because separating them would have produced strata too small to support stable estimation. Both the reliance on surgeon-reported EOR and the pooling of biopsy with partial resection are revisited in the Limitations.

#### 2.3.4. Postoperative Imaging Variables

We conducted a review of postoperative MRIs (performed consistent with institution protocol) to determine the following characteristics of residual tumor, assessed as binary (presence/absence) and, where measurable, as continuous variables. Residual tumors were defined as any visual presence of identifiable tumor tissue on early postoperative imaging. The rationale for including imaging-derived residual tumor metrics was to account for possible discrepancies between intraoperative evaluations of tumor size/volume and the imaging-derived residual tumor measurement.

Imaging protocol relied on the standard brain tumor MRI sequences routinely used in clinical practice, which included T1-weighted sequences acquired before and after gadolinium administration, T2-weighted imaging, fluid-attenuated inversion recovery (FLAIR), and susceptibility-weighted imaging (SWI). Additional sequences were obtained selectively, depending on the clinical context.

The evaluated imaging parameters included the presence or absence of residual tumor, contrast-enhancing components, and other structural abnormalities detected on the first post-treatment MRI. Postoperative hemorrhage was defined as any susceptibility-related signal abnormality visible on SWI, including small or clinically subtle findings; this deliberately inclusive definition is sensitive but not specific for clinically significant postoperative bleeding. These imaging features were entered into the statistical models as independent variables and were assessed for their potential association with RFS and the other clinical outcomes of interest. All imaging assessments were performed as routine clinical care and were subsequently curated within the institutional research database used for the present analysis.

#### 2.3.5. Treatment Variables

Adjuvant treatment data included postoperative radiotherapy (yes/no), recorded as a binary variable. Radiotherapy was entered among the candidate covariates in multivariable models to account for its potential modifying effect on recurrence risk but was not retained in the final models after AIC-based refinement, reflecting its near-uniform delivery in this cohort (259 of 281 patients, 92.2%), which leaves little between-patient variation to exploit. First-line temozolomide was recorded but was not entered as a covariate: its administration was determined almost entirely by histological subtype and WHO grade, both of which were already included in the models, so that adding it would have introduced substantial redundancy without contributing independent information. The absence of a chemotherapy term is acknowledged in the Limitations. When available, treatment patterns were interpreted in the context of standard-of-care protocols.

#### 2.3.6. Variable Naming and Coding

For clarity and consistency, variables are reported in the manuscript using descriptive clinical labels rather than database field names. The primary outcomes were 1-year recurrence-free survival (RFS_1) and 2-year recurrence-free survival (RFS_2). For these endpoints, a value of 1 indicated that the patient remained recurrence-free during the specified follow-up interval, whereas 0 indicated recurrence within that interval.

Secondary outcomes were overall recurrence, reoperation after recurrence, and reirradiation after recurrence. The three EOR categories (GTR, STR, PR) are defined in Section 2.3.3. KPI values were analyzed as continuous variables. When binary outcomes were summarized descriptively, 1 indicated presence of the outcome and 0 indicated absence of the outcome.

### 2.4. Outcome Measures

The primary endpoints were RFS at (i) 1 year after surgery (RFS_1) and (ii) 2 years after surgery (RFS_2). RFS was defined as the absence of radiological or histopathological evidence of tumor recurrence within the specified time interval. Recurrence was determined based on follow-up imaging and/or histopathological confirmation in cases of reoperation, in accordance with routine clinical practice and contemporary neuro-oncology standards. Response and progression were assessed using the Response Assessment in Neuro-Oncology (RANO) criteria as applied in routine practice at the treating institution [6]. Early postoperative MRI provided the post-surgical baseline, and follow-up MRI was obtained at short intervals thereafter. All follow-up examinations included perfusion sequences, which were used to help separate true recurrence from treatment-related change. Equivocal findings, particularly new or enlarging enhancement appearing within the first three months after chemoradiotherapy, were re-imaged before being classified, and cases that remained uncertain were adjudicated in the multidisciplinary neuro-oncology board. Histopathological confirmation was available in patients who proceeded to reoperation. Because this pathway reflected routine clinical practice and was not governed by a prespecified research protocol, a residual degree of misclassification cannot be excluded, and this is acknowledged in the Limitations.

For regression analyses, the 1-year and 2-year RFS endpoints were treated as binary variables, with a value of 1 indicating that the patient remained recurrence-free within the respective follow-up interval and a value of 0 indicating recurrence during that interval. Other endpoints that were assessed included: (1) reoperation, which was defined as any surgical intervention done after recurrence; (2) reirradiation, which was defined as any further radiation therapy delivered after the tumor recurs. The landmark formulation of RFS presupposes a postoperative state against which subsequent recurrence can be judged. After biopsy or partial resection, macroscopic tumor persisted at the end of the procedure by design, so a recurrence-free interval was not definable, and these patients were recorded as not evaluable for RFS_1 and RFS_2 rather than being assigned an event. This is a definitional property of the endpoint and not a form of attrition; its magnitude is quantified in Section 2.6, and its consequences for the resection comparison are examined in the Limitations. The overall recurrence endpoint carries no such requirement and was therefore assessable across essentially the whole cohort.

### 2.5. Statistical Analysis

Statistical analyses were performed using the R software (R version 4.5.1, R Foundation for Statistical Computing, Vienna, Austria) [18]. All analyses were performed in R, using dedicated packages for penalized logistic regression, model selection, nomogram construction, calibration, graphical visualization, and decision curve analysis. The software environment and packages used in the final analytical workflow are listed in Supplementary Table S2.

Categorical variables were summarized as absolute frequencies and percentages, with denominators based on evaluable cases (excluding patients with missing outcome data for the variable in question). Continuous variables were summarized as appropriate to their distribution.

The primary outcomes—1-year and 2-year recurrence-free survival (RFS_1, RFS_2)—and the secondary outcomes—overall recurrence, reoperation after recurrence, and reirradiation after recurrence—were analyzed as binary endpoints. For RFS endpoints, the event of interest corresponded to remaining recurrence-free within the specified follow-up window.

Univariable logistic regression was used to assess crude associations between candidate explanatory variables and each outcome, with results expressed as odds ratios (ORs) with 95% confidence intervals (CIs). To address the limited sample size and the potential for quasi-complete or complete separation in subgroups defined by the categorical predictors, Firth’s penalized logistic regression was used for both the univariable and the subsequent multivariable models. Firth’s correction reduces small-sample bias and provides finite estimates in the presence of separation, while still allowing classical hypothesis testing.

For each outcome, multivariable models were constructed by combining variables identified as clinically relevant a priori with those showing meaningful univariable associations. Model refinement was guided by the Akaike Information Criterion (AIC) within a stepwise procedure, and multicollinearity was assessed before finalizing each model. Extent of resection and postoperative residual tumor were examined jointly before both were retained: the two are conceptually related but not redundant, since residual tumor was recorded from imaging independently of the surgeon-reported resection category, and no indication of collinearity emerged that would have precluded keeping both terms in the models. Extent of resection was modeled as a categorical variable with partial resection/biopsy (PR) as the reference, and histological subtype was modeled with oligodendroglioma as the reference. Although Firth’s correction provides stable point estimates under separation, the resulting confidence intervals can be wide and the corresponding effect sizes should be interpreted as indicating direction and approximate magnitude rather than precise effect estimates; this point is revisited in the Limitations.

For the overall recurrence outcome, exploratory predictive modeling, including a nomogram with internal calibration and decision curve analysis, was additionally performed and is reported in Supplementary Materials.

All analyses followed a complete-case approach (see Section 2.6). A two-sided *p*-value < 0.05 was considered statistically significant.

The choice of binary landmark models over time-to-event analysis warrants explanation. Recurrence status was ascertained from clinical and imaging follow-up at fixed landmark intervals rather than as an exact event date, and the spacing of surveillance examinations varied with clinical need. Kaplan–Meier estimation and Cox proportional hazards regression require event times that are both accurate and uniformly ascertained; applied to landmark-derived, interval-censored status they would have conveyed a temporal resolution the data do not possess. A binary landmark formulation at 1 and 2 years was therefore prespecified. The cost is that the timing information within each interval is not exploited and that each analysis is confined to patients in whom the landmark could be reached, and this trade-off is revisited in the Limitations.

### 2.6. Missing Data Handling

Missing data were handled using a complete-case approach for each outcome model: patients with missing values for the outcome of interest were excluded from the corresponding analysis, and denominators in descriptive statistics reflect evaluable cases only. The number of patients in whom each outcome could not be evaluated is reported transparently in Table 1.

The proportion of unavailable outcome data varied by endpoint. It reflects the structure of the underlying clinical follow-up and, for the landmark endpoints, the definition of the endpoint itself; it does not stem from systematic data entry gaps. For the primary RFS endpoints (RFS_1, RFS_2), 96 of 283 patients (33.9%) could not be classified. This group is not distributed evenly across resection categories: RFS was determinable in 94 of 94 patients after GTR and in 77 of 77 after STR, but in only 16 of 100 after partial resection or biopsy, and in none of the 12 patients whose resection category was not documented. As set out in Section 2.4, this concentration follows from the endpoint rather than from differential loss to follow-up, since after a non-curative procedure residual macroscopic disease was present from the outset and a recurrence-free interval could not be defined. Recording these patients as not evaluable is the conservative option: had they instead been classified as events, the apparent advantage of GTR over the reference category would have been larger, not smaller. The practical consequence is that the reference stratum contributes few evaluable observations to the RFS models, which is reflected in the width of the corresponding confidence intervals and is addressed in the Limitations. For overall recurrence, an endpoint that requires no disease-free postoperative baseline and was therefore assessable across essentially the whole cohort, missingness was low (24/283; 8.5%). For the conditional secondary endpoints—reoperation and reirradiation after recurrence—denominators were further restricted to patients in whom recurrence was documented and treatment decisions could be retrieved (134/283 evaluable; 149 missing).

Multiple imputation was not undertaken, and the reasons differ by endpoint. At the landmark RFS endpoints, non-evaluability is predominantly definitional and not a missing value: in a patient in whom macroscopic tumor remained at the end of surgery, there is no underlying recurrence-free interval to recover, so imputation would fabricate an outcome instead of restoring one. For the conditional secondary endpoints, reoperation and reirradiation are defined only in patients with documented recurrence, so the restriction is structural. Where genuine incompleteness of follow-up does contribute, it is unlikely to be missing at random conditional on observed covariates, and imputation under such conditions could introduce more bias than it removes. The impact of non-evaluability on inference is therefore acknowledged in the Limitations section, and the interpretation of secondary endpoints is correspondingly cautious.

## 3. Results

### 3.1. Distribution of Clinical Outcomes

A total of 283 patients were included in the final analysis. Their baseline demographic, histopathological, surgical and treatment characteristics are summarized in Table 2, and the overall distribution of binary primary and secondary outcomes, together with the number of cases in whom the outcome could not be evaluated, is presented in Table 1. For all binary endpoints, percentages were calculated using the number of evaluable cases for the respective variable, excluding cases in which the outcome was not evaluable.

Tumor laterality and gross anatomical location were also recorded and were entered as covariates in the exploratory secondary analyses (Supplementary Section S3). As shown in the lower part of Table 2, a complete WHO 2021 integrated molecular profile was achievable in only a minority of patients, and MGMT promoter methylation was not assessed at all during the study period.

For the primary outcomes, among patients with available 1-year follow-up data, 70.6% (*n* = 132) remained recurrence-free, whereas 29.4% (*n* = 55) developed recurrence. At 2 years, among patients with available follow-up data, RFS decreased to 64.2% (*n* = 120), while 35.8% (*n* = 67) experienced disease progression. Outcome data were unavailable for 96 patients for both RFS_1 and RFS_2, as detailed in Table 1.

For the secondary outcomes, tumor recurrence was documented in 53.7% (*n* = 139) of evaluable patients, whereas 46.3% (*n* = 120) had no recorded recurrence. Reoperation was performed in 31.3% (*n* = 42) of patients with available data, while 68.7% (*n* = 92) did not undergo repeat surgery. Reirradiation was required in 35.1% (*n* = 47) of evaluable patients, whereas 64.9% (*n* = 87) did not receive additional radiotherapy. Missing data were limited for recurrence status (*n* = 24) but were substantial for reoperation and reirradiation (*n* = 149 for each endpoint), and this should be considered when interpreting these findings.

**Figure 1:**
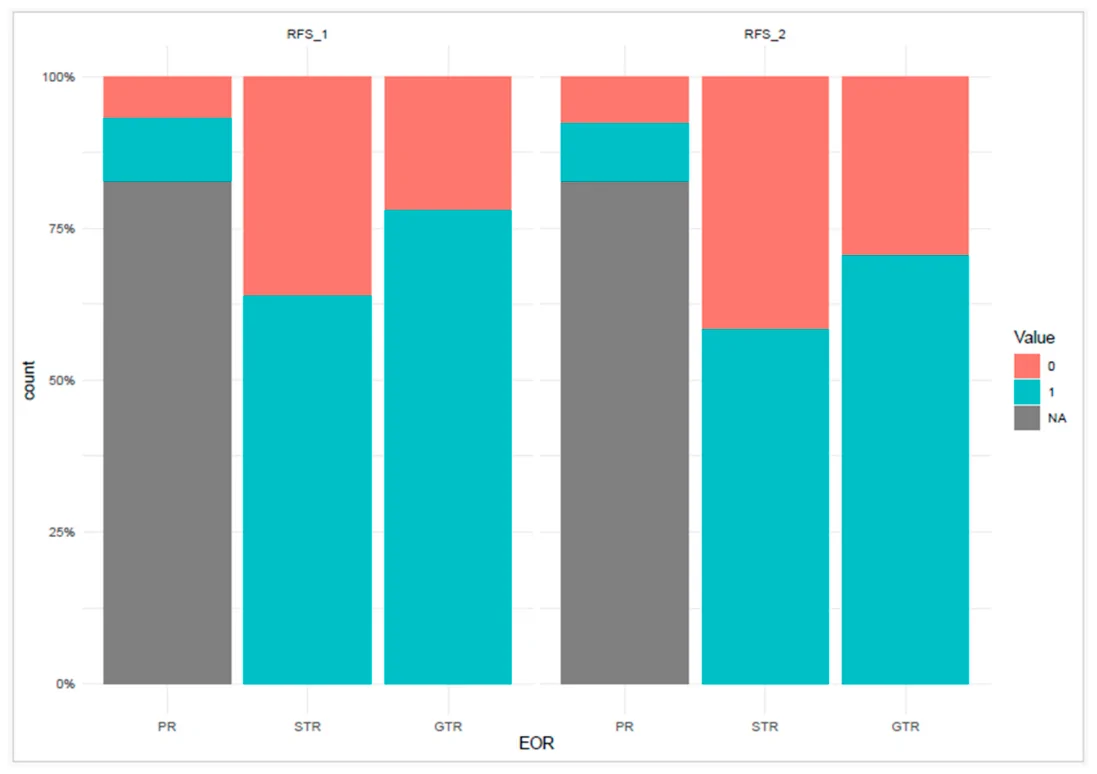
Distribution of recurrence-free survival according to extent of resection. Stacked bar plots showing RFS_1 and RFS_2 by extent of resection (EOR) (PR, partial resection or biopsy; STR, subtotal resection; GTR, gross total resection). A value of 1 indicates recurrence-free survival, 0 indicates recurrence/progression, and NA indicates that the endpoint was not evaluable. Patients in the NA category were excluded from all inferential models and were not imputed. The predominance of NA in the PR category is definitional rather than a consequence of loss to follow-up: after a non-curative procedure macroscopic tumor remained in situ, so a recurrence-free interval could not be defined (Section 2.4 and Section 2.6).

Evaluability for the landmark RFS endpoints differed markedly across resection categories, for the definitional reason set out in Section 2.4 and Section 2.6: RFS could be determined in all 94 patients after GTR and all 77 after STR, but in only 16 of 100 after PR. Figure 1 displays this non-evaluable fraction explicitly, and the resection comparison at these two endpoints should be read in that light. The distribution of RFS according to the extent of resection is shown in Figure 1. At both 1 and 2 years, GTR was associated with the highest proportion of recurrence-free patients, followed by STR, whereas PR showed the lowest proportion of RFS. Although RFS declined over time across all resection categories, the relative advantage of GTR remained apparent at the later time point.

The distribution of secondary clinical outcomes according to extent of resection is presented in Supplementary Figure S6. GTR was associated with a lower proportion of recurrence compared with PR and STR. By contrast, no clear separation between resection groups was observed for reoperation or reirradiation, both of which occurred relatively infrequently among evaluable cases. Notably, the overall recurrence endpoint was evaluable in 259 of 283 patients (91.5%) and shows the same ordering across resection categories as the landmark endpoints, despite a missing-data structure that is entirely different.

### 3.2. Univariable Analysis

The associations between extent of resection and each clinical outcome were first explored using Firth-penalized univariable logistic regression. For the 1-year and 2-year RFS endpoints, the unadjusted models did not reveal statistically significant differences across resection categories, with confidence intervals consistent with both protective and neutral effects. This pattern is informative rather than null: in retrospective glioma cohorts, the unadjusted association between extent of resection and RFS is frequently obscured by confounding from histological subtype and disease distribution, since more aggressive tumors tend to be both less amenable to complete resection and intrinsically more recurrence-prone. The independent contribution of resection therefore typically becomes apparent only after adjustment, as confirmed in our subsequent multivariable models (Section 3.3.1).

For overall recurrence, by contrast, the univariable model already showed a significant protective effect of GTR (OR 0.38, 95% CI 0.21–0.69, *p* = 0.001), while STR did not reach statistical significance.

For reoperation after recurrence, neither STR (OR 1.15, *p* = 0.8) nor GTR (OR 1.96, *p* = 0.2) was significantly associated with the outcome. Similarly, no statistically significant associations were identified between extent of resection and reirradiation after recurrence (STR: OR 1.18, *p* = 0.8; GTR: OR 2.35, *p* = 0.10). Overall, these findings suggest that univariable analyses capture only part of the signal associated with increasing extent of resection and do not fully account for clinically relevant confounding related to tumor histology, disease distribution, postoperative status, and imaging-defined residual disease.

### 3.3. Multivariable Analysis

**Figure 2:**
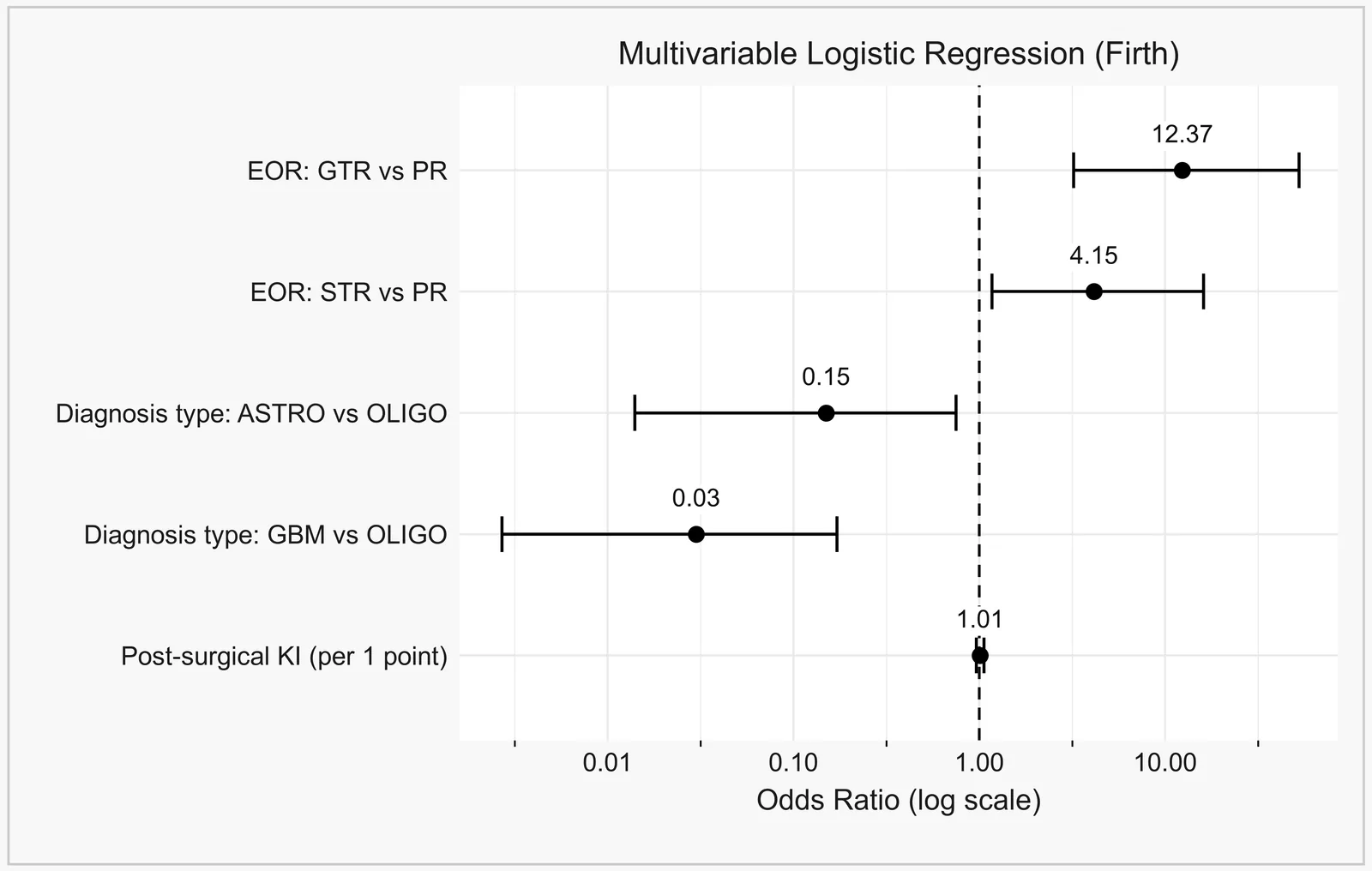
Forest plot of the multivariable logistic regression model (Firth correction) for recurrence-free survival at 1 year. Adjusted odds ratios and 95% confidence intervals from the Firth-corrected multivariable model are shown. Odds ratios greater than 1 indicate higher odds of remaining recurrence-free at 1 year. PR served as the reference category for extent of resection, and oligodendroglioma for histological subtype. KPI = Karnofsky Performance Index.

**Figure 3:**
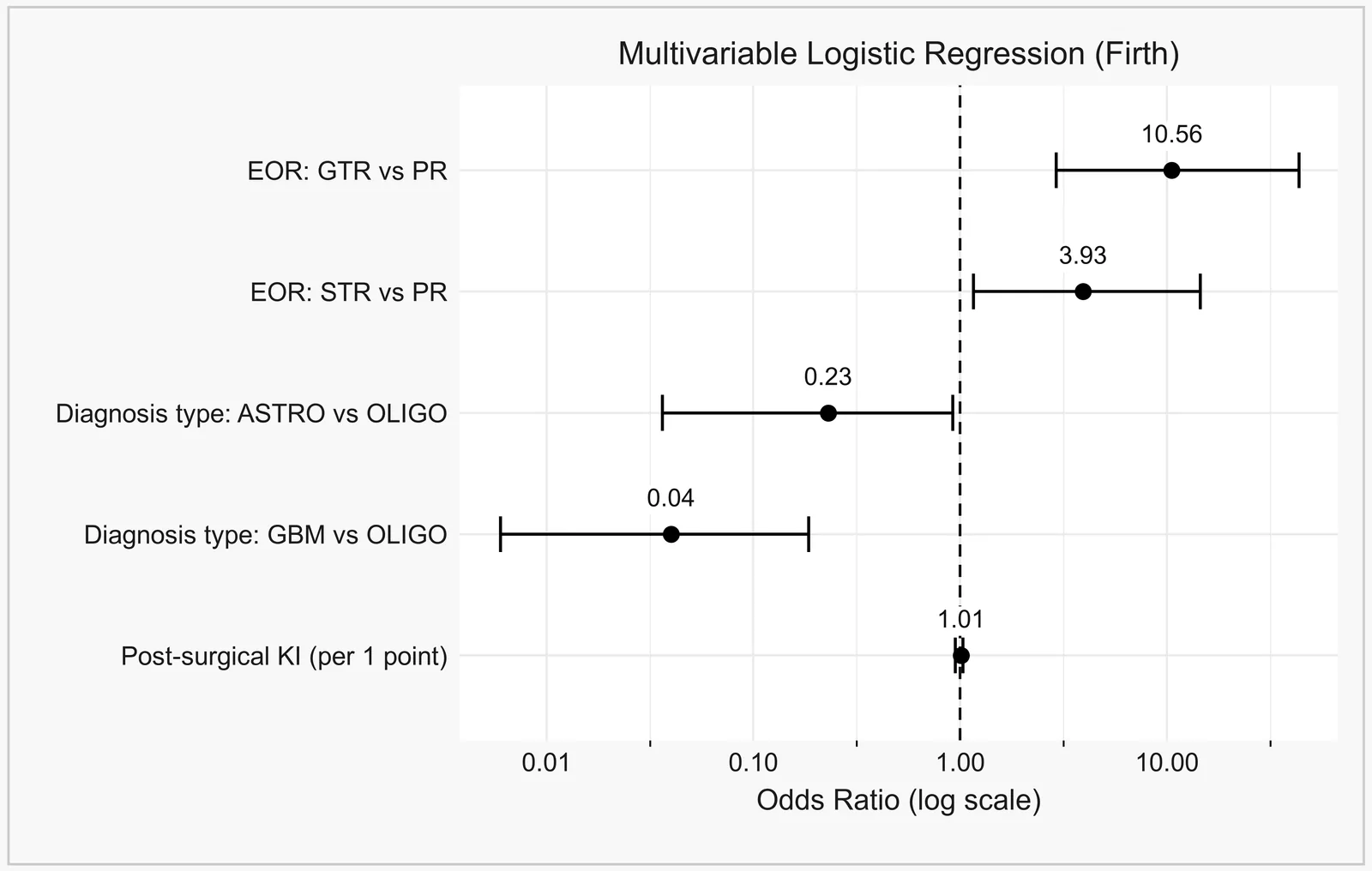
Forest plot of the multivariable logistic regression model (Firth correction) for recurrence-free survival at 2 years. Adjusted odds ratios and 95% confidence intervals from the Firth-corrected multivariable model. Odds ratios greater than 1 indicate higher odds of remaining recurrence-free at 2 years. PR served as the reference category for extent of resection, and oligodendroglioma for histological subtype. ASTRO = Astrocytoma; OLIGO = Oligodendroglioma; GBM = Glioblastoma; KPI = Karnofsky Performance Index.

**Figure 4:**
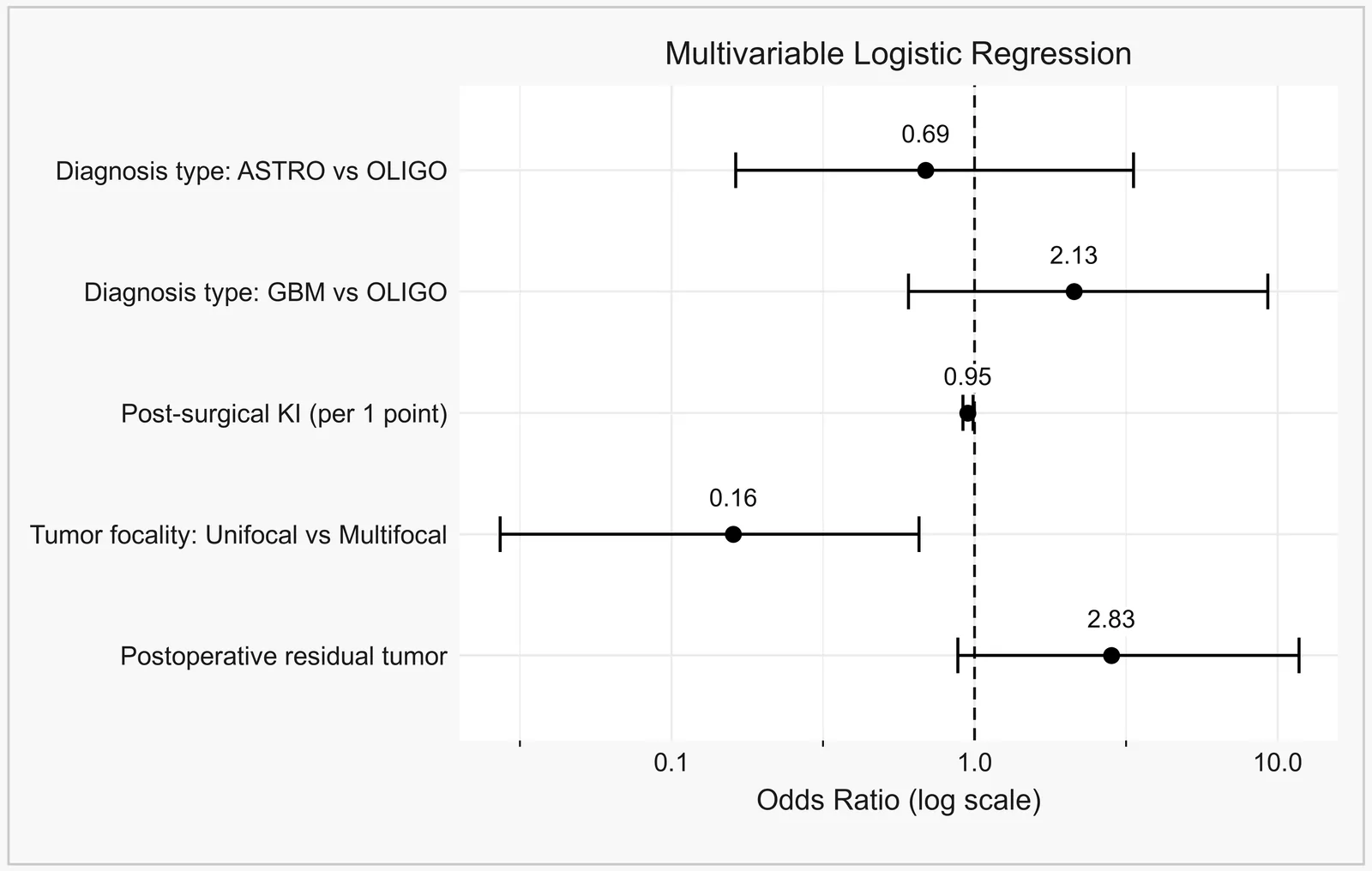
Multivariable logistic regression model for tumor recurrence. Adjusted odds ratios and 95% confidence intervals from the multivariable logistic regression model. Oligodendroglioma served as the reference category for histological subtype, and multifocal presentation as the reference category for tumor focality; the plotted focality estimate therefore expresses the odds of recurrence for unifocal relative to multifocal tumors, and an odds ratio below 1 indicates lower odds of recurrence.

To address potential confounding factors, multivariable logistic regression models were constructed for each outcome (Figure 2, Figure 3 and Figure 4).

#### 3.3.1. Recurrence-Free Survival

In the multivariable model for 1-year RFS, both GTR (OR 12.37, 95% CI 3.23–52.58, *p* < 0.001) and STR (OR 4.15, 95% CI 1.17–16.16, *p* = 0.027) were independently associated with higher odds of remaining recurrence-free compared with PR. Tumor histology also had a significant impact, with glioblastoma showing lower odds of 1-year RFS compared with oligodendroglioma (OR 0.03) and astrocytoma likewise showing lower odds than oligodendroglioma (OR 0.15); postoperative Karnofsky Index showed no independent association at this time point (OR 1.01) (Figure 2).

Similarly, in the multivariable model for 2-year RFS, GTR (OR 10.56, *p* < 0.001) and STR (OR 3.93, *p* = 0.027) remained significant independent predictors of higher odds of remaining recurrence-free compared with PR, confirming the sustained benefit of more extensive cytoreduction over time (Figure 3). Histological subtype remained a strong modifier at this time point as well: glioblastoma carried markedly lower odds of remaining recurrence-free at 2 years than oligodendroglioma (OR 0.04), and astrocytoma also carried lower odds (OR 0.23), while postoperative Karnofsky Index again showed no independent association (OR 1.01). Across both time points, the subtype of the tumor and the completeness of its removal behave as largely independent determinants of early recurrence: the two act in the same direction, and neither effect is explained away by the other once both are in the model.

#### 3.3.2. Recurrence

In the multivariable model for recurrence, postoperative functional status and tumor focality emerged as relevant determinants. A higher postoperative Karnofsky Index was associated with lower recurrence risk (OR 0.95, *p* = 0.015), whereas unifocal presentation carried markedly lower odds of recurrence than multifocal disease (unifocal relative to multifocal, OR 0.16). Postoperative residual tumor showed a non-significant trend toward increased recurrence risk (OR 2.83, with a confidence interval crossing unity), and in this model, histological subtype was not independently associated with recurrence (astrocytoma versus oligodendroglioma, OR 0.99; glioblastoma versus oligodendroglioma, OR 2.13; both with confidence intervals crossing unity) (Figure 4). The recurrence model therefore behaves differently from the RFS models: subtype governs how quickly recurrence occurs within the landmark windows, whereas over the whole observation period, functional status and disease distribution dominate whether recurrence is recorded at all.

Exploratory secondary analyses for the conditional outcomes of reoperation and reirradiation after recurrence are presented in Supplementary Materials (Section S3, Supplementary Figures S4 and S5). Briefly, age and tumor laterality predicted reoperation, while STR and younger age were associated with reirradiation; the small number of events and wide confidence intervals, particularly for reirradiation, constrain interpretation, and these results are best regarded as hypothesis-generating.

The multivariable findings for the overall recurrence outcome were additionally used to construct an exploratory recurrence-prediction nomogram with internal calibration and decision curve analysis, contextualized against published prognostic models in glioma; full results are provided in Supplementary Materials (Section S2, Supplementary Figures S1–S3). This nomogram is strictly exploratory and hypothesis-generating: it was internally derived from a single-center dataset with substantial missingness and has not been externally validated, and it is therefore not intended to guide individual treatment decisions in its current form.

## 4. Discussion

In this single-center cohort of 283 adults with surgically treated diffuse gliomas from a Romanian tertiary neuro-oncology center, GTR was independently associated with improved RFS at both 1 and 2 years, even though no significant effect emerged in the unadjusted analyses for these endpoints. To our knowledge, this is one of the few integrated multivariable evaluations of EOR-related recurrence dynamics, combining surgical, postoperative imaging, and clinical variables, reported from an Eastern European tertiary neuro-oncology setting. The analysis is not a report of therapeutic or technical advance, and we make no claim of methodological novelty. It quantifies the contribution of an established surgical objective under conditions of routine care in a setting that is rarely represented in the surgical glioma literature, and it should be judged on that basis. The findings illustrate, in a contemporary cohort treated under the WHO 2021 framework, how the prognostic signal of maximal cytoreduction emerges only after appropriate adjustment for tumor biology and disease distribution.

The divergence between unadjusted and adjusted estimates aligns with a recurring pattern in retrospective glioma cohorts: tumor histology and disease distribution mask the surgical effect when not accounted for. Once these biological confounders are introduced, the contribution of surgical radicality becomes interpretable, consistent with the integrated histomolecular framework of WHO 2021 [1,2].

Our results echo the trajectory of the recent surgical-oncology literature in adult-type diffuse glioma. Molinaro et al. linked maximal resection of both contrast-enhancing and non-enhancing components to prolonged survival across molecular subgroups in glioblastoma [4], while Karschnia et al. proposed the standardized EOR categories now adopted by the RANO resect group, which frames resection as a biologically and prognostically relevant variable within a multimodal framework rather than a binary operative achievement [3,5]. The substantial adjusted effect of GTR in our cohort, observable only after accounting for histology and postoperative status, fits this framework.

Our cohort differs in instructive ways from series reported in settings where diagnostic resources are less constrained, and the most visible difference is diagnostic, not surgical. In our patients, 1p/19q codeletion was assessed in 23.3% of cases, and MGMT promoter methylation was not tested routinely at all. The Western European and North American cohorts that underpin current EOR recommendations, such as the University of California San Francisco series analyzed by Molinaro et al. [4] and the multicenter cohorts used to derive and validate the RANO resect categories [5,19], report near-complete molecular annotation and, in several instances, volumetric tumor segmentation. The distribution of EOR also differs. GTR was achieved in 34.7% of patients with a documented resection category in our series, a lower proportion than is reported from centers with routine access to intraoperative MRI, 5-aminolevulinic acid fluorescence guidance and awake mapping. In our data the direction and the independence of the EOR effect nevertheless survive this gap: the association between more complete resection and lower early recurrence is recoverable in a setting where the biological covariates that Western series adjust for are largely unavailable, and where the surgical adjuncts that facilitate radical resection are unevenly distributed. The observation matters because guideline recommendations formulated in well-resourced centers are applied in centers such as ours, and evidence that they remain directionally valid under these conditions has been scarce [12,13].

A further question concerns why a given resection category was achieved at all. The objective of surgery in diffuse glioma is removal of the entire imaging-defined tumor, and the principal constraint on achieving it is the relationship between tumor and functional cortical and subcortical tissue, a trade-off between surgical radicality and functional outcome that has been quantified in low-grade glioma [20]. Tumors in or adjacent to eloquent territory are systematically less likely to be resected completely, and they may also differ prognostically for reasons that have nothing to do with surgery, so part of the association reported here may reflect surgical accessibility rather than the act of resection itself. Laterality and gross anatomical location were recorded and appeared in the secondary models, but involvement of eloquent areas was not systematically coded, and the availability of intraoperative mapping and awake craniotomy varied over the study period. EOR in this cohort is therefore best understood as a composite of surgical intent, surgical technique and tumor location, whose components we cannot fully separate.

The effect of EOR persisted at both 1 and 2 years, suggesting that maximal cytoreduction confers durable rather than only immediate benefit. This temporal pattern is consistent with refined EOR classifications validated by Karschnia et al. in glioblastoma [19], and with evidence that molecular subgroups within IDH-wildtype glioblastoma derive differential benefit from aggressive resection [21], both reinforcing the interaction between cytoreduction and tumor biology that emerges most clearly in adjusted analyses.

Histology emerged as a strong RFS modifier in our models, with astrocytoma and glioblastoma carrying lower RFS odds than oligodendroglioma. The pattern aligns with the joint role of EOR and molecular subtype demonstrated by Choi et al. in WHO grade II glioma [9] and by Garton et al. in 1p/19q-codeleted tumors [22], and with the broader argument that the benefit of resection in low-grade glioma must be read against the molecular signature, not morphology alone [10].

In our cohort, postoperative residual tumor showed a trend toward increased recurrence risk that did not reach significance across all multivariable models, likely because the categorical EOR variable already absorbs much of the prognostic information carried by residual burden. The wider literature continues to support residual disease as a prognostic factor: Incekara et al. linked minimal residual contrast-enhancing and non-enhancing tumor to improved survival in IDH-wildtype glioblastoma [8], and Skardelly et al. described a continuous correlation between residual volume and survival [23]. Our findings are compatible with this view, with the EOR threshold capturing the dominant signal in our setting.

In the recurrence model, unifocal tumors and a higher postoperative Karnofsky Index were independently protective. Multifocality likely captures both wider tumor dissemination and reduced surgical achievability, since complete macroscopic clearance is harder to attain—consistent with the poor prognosis and prognostic relevance of volumetric MRI parameters in multifocal glioblastoma reported by Kasper et al. [24]. Postoperative Karnofsky Index did not independently predict adjusted RFS but was associated with recurrence, plausibly reflecting better tolerance of timely adjuvant therapy in patients with preserved function.

Incomplete outcome data could in principle account for the surgical association we report, and the point requires a specific answer. Only the two landmark RFS endpoints are affected, and, for the definitional reason set out above, the effect falls almost entirely on the non-curative reference stratum: RFS was determinable in every patient after GTR and after STR, but in 16 of 100 after PR. The overall recurrence endpoint is not subject to that constraint. It requires no disease-free postoperative baseline, was evaluable in 259 of 283 patients (91.5%), and shows the same gradient, with GTR associated with lower odds of recurrence even in unadjusted analysis (OR 0.38, 95% CI 0.21–0.69, *p* = 0.001). Two endpoints with very different missing-data structures thus point the same way. Neither is decisive alone, but their concordance makes evaluability an unlikely sole explanation for the association.

Exploratory analyses of reoperation and reirradiation, presented in Supplementary Materials, are consistent with the established complexity of patient selection for repeat surgery [25,26] and re-irradiation [27] in recurrent high-grade glioma. Exploratory predictive modeling for the overall recurrence outcome is presented in Supplementary Materials, contextualized against published glioma nomograms [28,29,30].

The clinical implications of these findings are twofold. First, they reinforce that maximal safe resection should remain a central objective of diffuse glioma surgery, not simply because it improves overall survival in historical series, but because it appears to independently shape early recurrence dynamics in routine practice. Second, they indicate that routinely available clinical variables carry prognostic information not captured by the resection category alone: postoperative Karnofsky Index and tumor focality were independently associated with overall recurrence after adjustment, whereas imaging-defined residual tumor showed only a non-significant trend once EOR was in the model. This supports an integrated approach to recurrence risk assessment in routine neuro-oncology workflows [3], while leaving open whether postoperative MRI adds independent prognostic information beyond the resection category, a question that will require larger series with volumetric measurement of residual disease [3].

### Limitations and Strengths

Several limitations should be acknowledged. First, the retrospective, single-center design introduces the possibility of selection bias, residual confounding, and practice-pattern effects that may limit external generalizability. The patient cohort reflects referral patterns and surgical decision-making at a single Romanian tertiary neuro-oncology center, and the findings should be confirmed in independent cohorts before broader inferences are drawn.

Second, the cohort encompasses heterogeneous diffuse glioma entities (astrocytoma, oligodendroglioma and glioblastoma, spanning CNS WHO grades 2 to 4). While this heterogeneity reflects the real-world spectrum of cases managed in a tertiary neuro-oncology setting and supports the external validity of the findings, it also complicates direct comparisons across narrower biological subgroups. Formal subgroup analyses stratified by histological subtype were not undertaken for this reason: with 38 oligodendrogliomas in the cohort and correspondingly fewer events, subtype-specific models would have been unstable and, under Firth penalization, would have produced estimates too imprecise to interpret. Histological subtype was instead retained as a covariate in every multivariable model, which quantifies its independent contribution but does not establish whether the effect of resection differs in magnitude between subtypes, a question that requires a larger and preferably multicenter sample.

Third, comprehensive molecular data—including IDH mutation status, 1p/19q codeletion, and MGMT promoter methylation—were not uniformly available across the cohort, reflecting the progressive integration of molecular profiling into routine practice during the study period (2021–2024). As a result, molecular variables could not be incorporated as covariates in the multivariable models, restricting the precision of biologically stratified interpretation and leaving open the possibility of residual confounding by tumor biology. Histological subtype and CNS WHO grade were retained as covariates and act as partial proxies for the underlying molecular classes, but they are imperfect substitutes, and the association between EOR and recurrence reported here should be read as adjusted for biology only to the extent that these proxies capture it. Findings should therefore be regarded as describing recurrence dynamics in a clinically and radiologically defined cohort, with biological refinement to be addressed in future analyses.

Fourth, outcome data were unavailable for a substantial number of patients at some endpoints, and the reasons differ between endpoints in a way that matters for interpretation. For the conditional secondary outcomes (reoperation and reirradiation, 149 of 283 cases not evaluable), the restriction is structural, since these endpoints are defined only in patients with documented recurrence and retrievable treatment decisions. For the landmark RFS endpoints (96 of 283), the dominant reason is definitional and not attritional: after biopsy or partial resection, macroscopic tumor remained in situ, so a recurrence-free interval could not be defined, and 84 of these 96 patients belong to that stratum. The consequence is that the PR reference category contributes only 16 evaluable observations to the RFS models, which is the principal reason the corresponding odds ratios carry wide confidence intervals, and it means that the magnitude of the GTR and STR estimates at these endpoints should not be taken at face value. We did not formally compare baseline characteristics between evaluable and non-evaluable patients, and we cannot therefore rule out that the 16 evaluable partial-resection patients differ systematically from the remainder of that stratum; this is a genuine residual uncertainty. Two features of the data temper it. Recording these patients as not evaluable, instead of counting them as events, is the conservative choice, since classifying them as events would have widened the apparent advantage of GTR. And the overall recurrence endpoint, which is free of this definitional constraint and was evaluable in 259 of 283 patients, reproduces the same direction of effect. Analyses followed a complete-case approach throughout, and results for endpoints with substantial non-evaluability should be interpreted as exploratory.

Fifth, the exposure itself was measured imperfectly. EOR was derived primarily from the operative report and the surgeon-reported resection percentage, and not from a volumetric comparison of pre- and postoperative tumor volumes, which is the standard recommended for prospective work by the RANO resect group. Surgeon-reported EOR is known to be optimistic relative to volumetric assessment, and misclassification of this kind would tend to blur the contrasts reported here, not create them. Presence of residual tumor on early postoperative imaging was entered as a separate covariate to mitigate this, but it captures only whether residual disease was detectable, not the resection fraction it implies. Biopsy and partial resection were also pooled into a single reference stratum, and these are not equivalent procedures; they share only the property of leaving measurable disease in situ, and a larger series should keep them apart.

Sixth, the endpoint was analyzed in landmark binary form and not as time to event. Recurrence status was ascertained at fixed intervals rather than as an exact date, so Kaplan–Meier and Cox analyses would have implied a temporal precision the data do not support; the price is that information about when recurrence occurred within each interval is not used, and that patients who had not yet reached a landmark could not contribute to it. Separately, although recurrence was assessed using RANO criteria with perfusion sequences and multidisciplinary adjudication of equivocal findings, pseudoprogression was handled within routine clinical practice and not under a prespecified research protocol, so some residual misclassification of early treatment-related change as recurrence cannot be excluded.

Seventh, unmeasured confounding remains possible. Involvement of eloquent cortical and subcortical territory, which simultaneously constrains the achievable extent of resection and carries its own prognostic weight, was not systematically coded, so the reported association between EOR and recurrence may partly reflect tumor accessibility. First-line temozolomide was recorded but not entered as a covariate because its administration was determined almost entirely by subtype and grade, which are already in the models; the resulting absence of an explicit chemotherapy term means that any residual effect of systemic treatment beyond what subtype and grade capture is not accounted for.

Eighth, several adjusted estimates carry wide confidence intervals—most prominently for subtotal resection in the reirradiation model—reflecting quasi-complete separation in subgroups defined by categorical predictors. Although Firth’s penalized regression yields finite point estimates under such conditions, the corresponding effect sizes should be interpreted as indicating the direction of association rather than its precise magnitude. The recurrence-prediction nomogram, calibrated and assessed by decision curve analysis on the same dataset, also requires external validation before clinical implementation outside the present institutional context. It was internally validated only, and we regard external validation in an independent cohort as a prerequisite, not an optional refinement; we intend to pursue it within a multicenter collaboration, and until then the nomogram should be treated as a description of the present dataset and not as a clinical tool.

The study also has several strengths: a consecutive real-world cohort treated entirely under the WHO 2021 classification framework, a clinically intuitive set of routinely available variables, the use of penalized regression appropriate for the cohort size, and the inclusion of exploratory predictive modeling reported transparently in Supplementary Materials. These elements, together with the regional context, support the relevance of the findings while motivating prospective multicenter and molecularly refined validation in future work.

## 5. Conclusions

In this real-world Romanian single-center cohort, EOR was independently associated with RFS in diffuse gliomas when evaluated within a multivariable framework. GTR was associated with improved 1- and 2-year RFS, supporting maximal safe resection as a primary oncological objective in contemporary glioma surgery. The strength of that association should be read with the evaluability of the landmark endpoints in mind: these data establish the direction of the effect, not its magnitude.

Surgical outcomes, however, cannot be interpreted in isolation. Tumor histology, focality, postoperative functional status, and residual tumor burden also contributed to recurrence risk, supporting an integrated prognostic approach in which surgical, imaging, and clinical variables are considered jointly rather than sequentially.

Taken together, the findings reinforce the continued relevance of maximal safe resection in the WHO 2021 era and highlight the added value of combining routinely available surgical, clinical, and imaging variables in postoperative prognostic modeling. Prospective multicenter validation, including molecularly stratified subgroup analyses, is needed to refine these associations and to externally evaluate the exploratory predictive model presented in Supplementary Materials.

## Figures and Tables

**Table 1:** Distribution of binary clinical outcomes in the overall cohort.

Endpoint	Evaluable, *n*	Outcome Present (1), *n* (%)	Outcome Absent (0), *n* (%)	Not Evaluable, *n*
1-year recurrence-free survival (RFS_1)	187	132 (71)	55 (29)	96
2-year recurrence-free survival (RFS_2)	187	120 (64)	67 (36)	96
Overall recurrence	259	139 (54)	120 (46)	24
Reoperation after recurrence	134	42 (31)	92 (69)	149
Reirradiation after recurrence	134	47 (35)	87 (65)	149

Percentages are calculated from evaluable cases only. For the recurrence-free survival endpoints, 1 denotes remaining recurrence-free within the interval and 0 denotes recurrence; for the remaining endpoints, 1 denotes presence and 0 absence of the outcome. Patients recorded as not evaluable were excluded from the corresponding analyses and were not imputed. Non-evaluability at the RFS endpoints is largely definitional and is concentrated in the partial resection/biopsy stratum (84 of 96 cases; Section 2.4 and Section 2.6).

**Table 2:** Baseline demographic, histopathological, surgical and treatment characteristics of the study cohort (*n* = 283).

Characteristic	Value
Age at diagnosis, years, median (range)	47 (15–88)
Sex, female/male, *n* (%)	145 (51.2)/138 (48.8)
Histological category, *n* (%)	
Glioblastoma	136 (48.1)
Astrocytoma	109 (38.5)
Oligodendroglioma	38 (13.4)
CNS WHO grade, *n* (%)	
Grade 2	86 (30.4)
Grade 3	48 (17.0)
Grade 4	149 (52.7)
Tumor focality, *n* (%)	
Unifocal	250 (88.3)
Multifocal	33 (11.7)
Extent of resection, *n* (%) a	
Gross total resection (GTR)	94 (34.7)
Subtotal resection (STR)	77 (28.4)
Partial resection or biopsy (PR)	100 (36.9)
Not documented	12
Karnofsky Performance Index, median	
Preoperative	100
Postoperative	100
Postoperative radiotherapy, *n* (%)	259/281 (92.2)
First-line temozolomide, *n* (%)	160 (56.5)
Molecular marker assessed, *n* (%) b	
IDH status	244 (86.2)
ATRX	190 (67.1)
Ki-67	233 (82.3)
1p/19q codeletion	66 (23.3)
MGMT promoter methylation	Not routinely assessed

a There were 271 patients with a documented resection category, consistent with the denominators used throughout. b Percentages for molecular markers indicate the proportion of the whole cohort in whom the marker was assessed, not the proportion positive; IDH was mutant in 123 of the 244 tested patients (50.4%). Molecular testing was performed across several pathology laboratories without a uniform assay platform (Section 2.3.2).

## Data Availability

The original contributions presented in this study are included in the article/Supplementary Material. Further inquiries can be directed to the corresponding authors.

## Supplementary Materials

The following supporting information can be downloaded at: https://www.mdpi.com/article/10.3390/clinpract16080155/s1, Table S1: STROBE checklist for observational cohort studies; Table S2: R software environment and analysis packages used in the final workflow; Section S2: Exploratory predictive modeling; Figure S1: Exploratory nomogram for overall recurrence; Figure S2: Calibration plot; Figure S3: Decision curve analysis; Section S3: Exploratory secondary outcome analyses; Figure S4: Forest plot for reoperation; Figure S5: Forest plot for reirradiation; Figure S6: Distribution of secondary clinical outcomes according to extent of resection.
