# Supplementary material for: Gross Total Resection and Recurrence-Free Survival in Diffuse Gliomas: A Single-Center Cohort Study from Eastern Europe
Source: Clin Pract. 2026 Aug 20;16(8):155. doi: 10.3390/clinpract16080155 (PMC13510490; doi:10.3390/clinpract16080155)
Supplement: Supplementary file 1 [file clinpract-16-00155-s001.zip › clinpract-4378432-supplementary.pdf]

SUPPLEMENTARY MATERIALS

Supplementary Table S1. STROBE checklist for observational cohort studies

STROBE item	Recommendation	Location in manuscript
Title and abstract	Indicate the study design and provide a balanced summary of methods and results.	Title; Abstract
Background/rationale	Explain the scientific background and rationale.	Introduction
Objectives	State the specific objectives and prespecified hypotheses.	Introduction
Study design	Present key elements of study design early in the paper.	Section 2.1
Setting	Describe the setting, locations, and relevant dates.	Section 2.2
Participants	Give the eligibility criteria and sources/methods of selection.	Section 2.2
Variables	Clearly define outcomes, exposures, predictors, confounders, and effect modifiers.	Sections 2.3-2.4
Data sources/measurement	Describe data sources and methods of assessment.	Section 2.3
Bias	Describe efforts to address potential sources of bias.	Sections 2.2, 2.5, Discussion
Study size	Explain how the study size was arrived at.	Section 2.2
Missing data	Explain how missing data were addressed.	Section 2.6; Table 2; Discussion 4.1
Quantitative variables	Explain how quantitative variables were handled in the analyses.	Sections 2.3.6, 2.5
Statistical methods	Describe all statistical methods, including confounding control.	Section 2.5
Participants/results	Report numbers of individuals at each stage and outcome data.	Results; Tables 1 and 2

Outcome data	Report numbers of outcome events or summary measures.	Results; Table 2
Main results	Give unadjusted and adjusted estimates with precision.	Sections 3.2-3.3; Figures 2-4
Other analyses	Report additional analyses, including calibration and decision-curve analysis.	Supplementary Section S2; Figures S1-S3
Limitations	Discuss study limitations, including potential bias and imprecision.	Discussion
Interpretation	Give a cautious overall interpretation of the results.	Discussion; Conclusions
Generalisability	Discuss the external validity of the findings.	Discussion
Funding/ethics	Report funding sources, ethics approval, and conflicts of interest.	Front matter

Supplementary Table S2. R software environment and analysis packages used in the final workflow

Software/package	Purpose in the analysis	Reported in manuscript
R	Primary statistical computing environment	Section 2.5; Reference 19
logistf	Firth's penalized logistic regression	Section 2.5
MASS	Model refinement/AIC-based selection workflow	Section 2.5
rms	Regression modeling strategies, nomogram development, and calibration	Section 2.5
rmda	Decision curve analysis	Section 2.5
ggplot2	Graphical output and figure generation	Section 2.5

Section S2. Exploratory Predictive Modeling: Nomogram, Calibration, and Decision Curve Analysis

As an exploratory extension of the multivariable analyses, the model for overall recurrence was used to construct a nomogram for individualized risk estimation (Supplementary Figure S1). The nomogram incorporates the covariates of the multivariable recurrence model: extent of resection, histological subtype, tumor focality, postoperative Karnofsky Index, and postoperative residual tumor. Each variable contributes a weighted score proportional to its regression coefficient, allowing calculation of a total score that corresponds to an

individualized probability of recurrence. Multifocal tumor presentation and lower postoperative Karnofsky Index contributed substantially to the predicted recurrence profile, while extent of resection also influenced the estimated probability.

Internal model performance was assessed using a calibration curve (apparent and bias-corrected by bootstrap, Supplementary Figure S2), which demonstrated good agreement between predicted and observed recurrence probabilities across the spectrum of risk, with only slight overestimation in the highest-risk subgroup. Stratification of the cohort by predicted probability separated patients into low-, intermediate-, and high-risk groups, with observed recurrence frequencies of 18%, 41%, and 88%, respectively.

Decision curve analysis (Supplementary Figure S3) showed a favorable net benefit of the nomogram-based strategy compared with "treat-all" and "treat-none" reference strategies across a clinically relevant range of threshold probabilities, suggesting potential utility for identifying higher-risk patients who may benefit from closer surveillance or intensified adjuvant therapy.

Important interpretive caveat. These analyses are exploratory and require external validation before any clinical implementation can be considered. The nomogram was derived from the same cohort used to evaluate calibration and decision curve performance (apparent / bootstrap-corrected internal validation), and the DCA reflects training-data performance. The analyses are presented to contextualize our findings against published prognostic models in glioma (28–30) and to motivate prospective, multicenter, externally validated work, not as a clinically deployable tool. We regard external validation in an independent, preferably multicenter cohort as a prerequisite rather than an optional refinement, and we intend to pursue it before any clinical application of this model is proposed.

Supplementary Figure S1. Exploratory nomogram derived from the multivariable logistic regression model for prediction of overall recurrence.

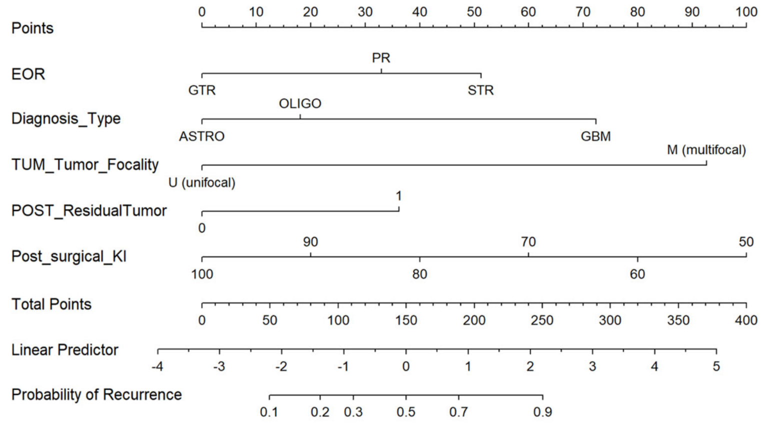

Each predictor is assigned a weighted score proportional to its regression coefficient, and the total score corresponds to the estimated probability of recurrence. Higher total scores indicate a higher predicted risk of recurrence. U = unifocal; M = multifocal.

Supplementary Figure S2. Calibration plot of the exploratory recurrence prediction model.

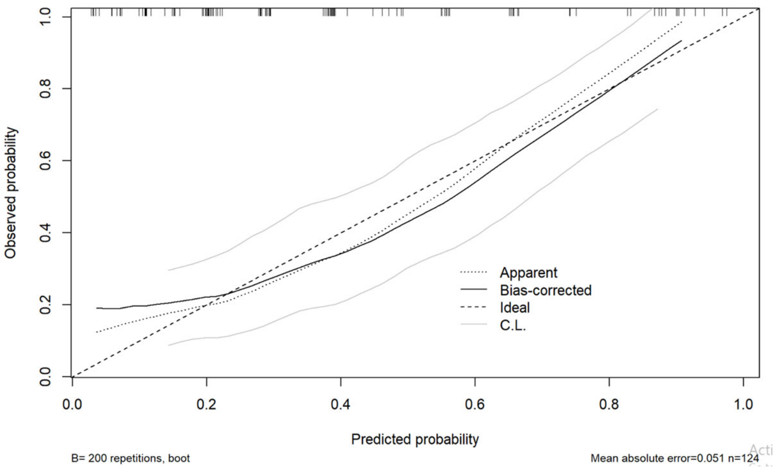

Calibration plot comparing predicted and observed probabilities of tumor recurrence. The diagonal line represents ideal calibration, while the apparent and bias-corrected curves show model performance across the range of predicted risk. ***C.I.*** *= confidence interval.*

Supplementary Figure S3. Decision curve analysis.

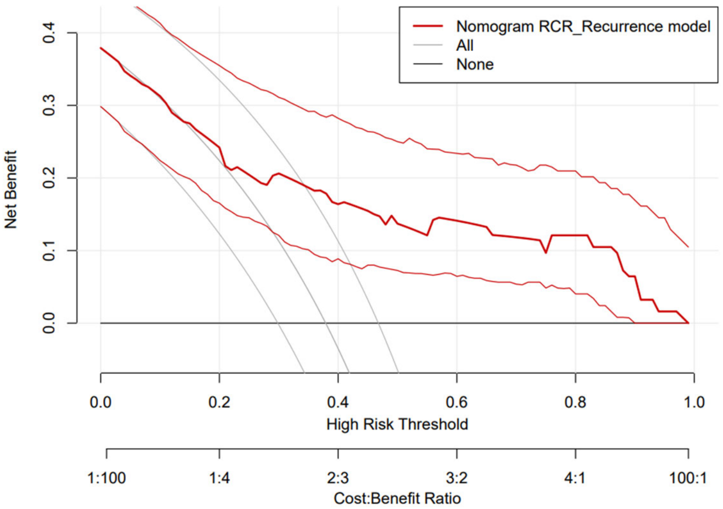

Decision curve analysis comparing the net benefit of the nomogram-based recurrence model with the default strategies of treating all patients or treating none across a range of threshold probabilities. The x-axis shows the threshold probability, and the y-axis shows net benefit.

Section S3. Exploratory Secondary Outcome Analyses: Reoperation and Reirradiation

As secondary endpoints, conditional outcomes of reoperation and reirradiation after recurrence were evaluated. Both analyses were limited to patients with documented recurrence and available retreatment information (n = 134 of 283 evaluable cases). Given the small number of events relative to the number of candidate predictors, these models are best regarded as exploratory and hypothesis-generating.

Reoperation. In the multivariable model for reoperation, age at diagnosis was an independent predictor (OR 0.95, p = 0.004), with younger patients more likely to undergo reoperation. Tumor laterality also demonstrated significant associations, particularly for right-sided and "other" locations. EOR did not independently influence reoperation rates (Supplementary Figure S4). This pattern is consistent with the established complexity of patient selection for repeat surgery in recurrent glioma. Yang et al. demonstrated in a matched cohort analysis that reoperation for recurrent glioblastoma remains controversial and highly dependent on patient selection [25], while Alhalabi et al. recently showed that repeat surgery may provide molecularly informative tissue and can be justified in the era of precision oncology when the risk–benefit balance is favorable [26]. Our observation that younger age predicted reoperation aligns with this literature: younger patients are more often selected for aggressive salvage treatment.

Reirradiation. In the multivariable model for reirradiation, subtotal resection was associated with markedly increased odds of reirradiation (OR 149.22, p = 0.004). The very wide confidence interval reflects quasi-complete separation in this subgroup — almost all reirradiated patients fell into a single resection category — and the point estimate should be interpreted as indicating direction rather than precise magnitude of effect. Age at diagnosis was inversely associated with reirradiation (OR 0.82 per additional year, p = 0.025), so that younger patients were more likely to be reirradiated, and ventricular tumor location was associated with lower reirradiation rates (Supplementary Figure S5). Given the small number of reirradiation events (n = 47) and the resulting model instability, this analysis is best interpreted as exploratory. The variable findings are consistent with the well-recognized complexity of reirradiation selection in clinical practice. Recent prospective data from Navarria et al. support the feasibility and efficacy of reirradiation in carefully selected patients with recurrent high-grade glioma [27]. Reirradiation is influenced by prior dose distributions, target volume, interval since first radiotherapy, patient performance status, and concomitant systemic strategies; retreatment decisions are often less determined by the initial resection category than by the pattern of recurrence and the therapeutic window available at progression.

Supplementary Figure S4. Forest plot of the multivariable logistic regression model for reoperation.

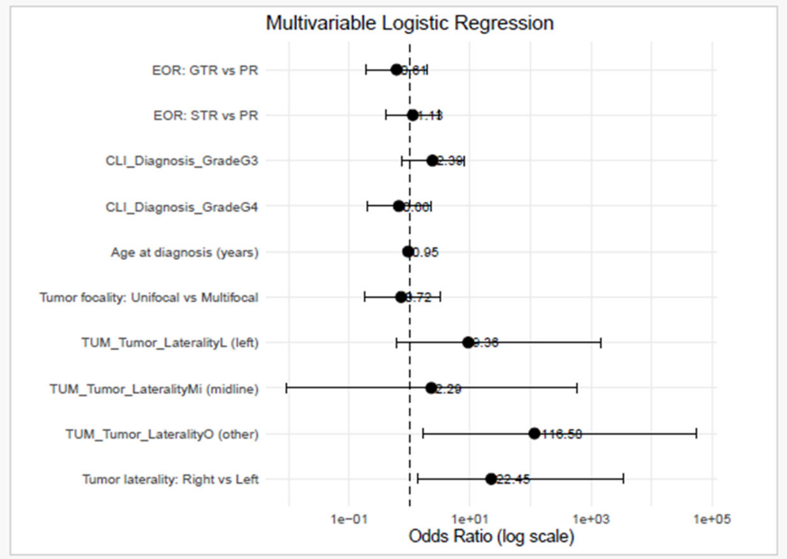

Adjusted odds ratios and 95% confidence intervals from the multivariable logistic regression model. ***PR*** *served as the reference category for extent of resection,* ***grade 2*** *as the reference category for histological grade,* ***unifocal*** *as the reference category for tumor focality, and* ***left-sided*** *laterality as the reference category for tumor laterality.*

Supplementary Figure S5. Forest plot of the multivariable logistic regression model for reirradiation.

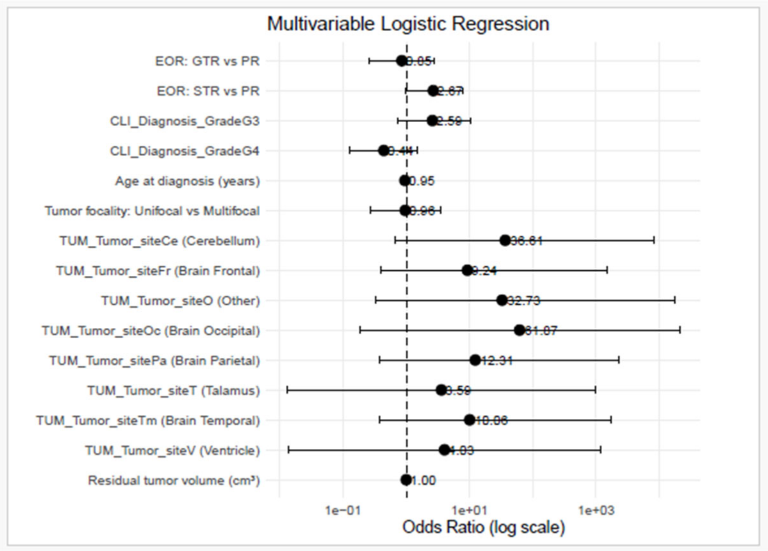

Adjusted odds ratios and 95% confidence intervals from the multivariable logistic regression model. ***PR*** *served as the reference category for extent of resection,* ***grade 2*** *as the reference category for histological grade,* ***unifocal*** *as the reference category for tumor focality, and the reference categories for tumor site are indicated in the forest plot labels. Residual tumor volume was analyzed as a continuous variable.*

Supplementary Figure S6. Distribution of secondary clinical outcomes according to extent of resection.

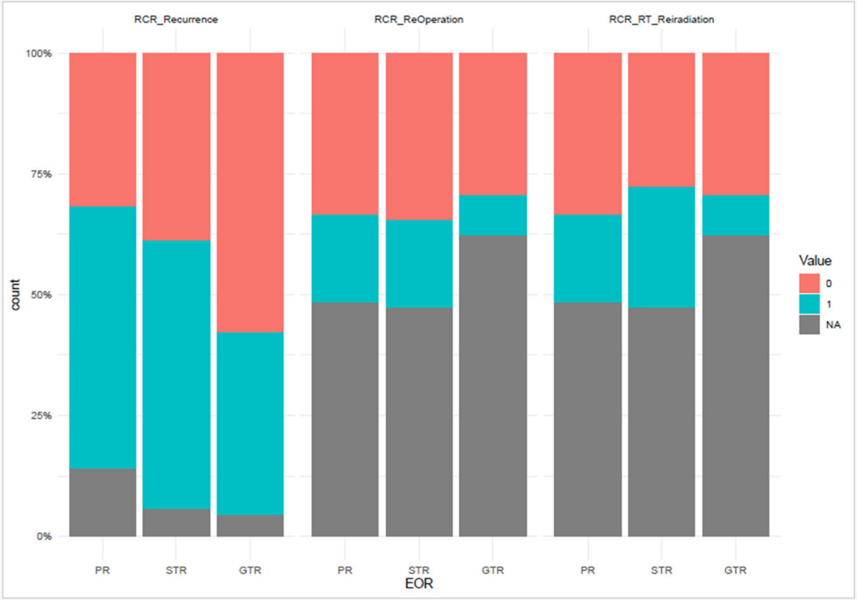

Stacked bar plots showing overall recurrence (RCR_Recurrence), reoperation after recurrence (RCR_Reoperation), and reirradiation after recurrence (RCR_RT_Reirradiation) by extent of resection (PR, partial resection or biopsy; STR, subtotal resection; GTR, gross total resection). For each endpoint, 1 indicates that the outcome occurred, 0 indicates that it did not, and NA indicates that the outcome could not be evaluated. Patients in the NA category were excluded from the corresponding analyses and were not imputed. Non-evaluability is limited for overall recurrence (24 of 283 patients) but substantial for the two conditional endpoints, which are defined only in patients with documented recurrence and retrievable retreatment decisions.
